# Chemical Biology Drug Sensitivity Screen Identifies Sunitinib as Synergistic Agent with Disulfiram in Prostate Cancer Cells

**DOI:** 10.1371/journal.pone.0051470

**Published:** 2012-12-12

**Authors:** Kirsi Ketola, Olli Kallioniemi, Kristiina Iljin

**Affiliations:** 1 VTT Technical Research Centre of Finland, and Turku Centre for Biotechnology, University of Turku, Turku, Finland; 2 Institute for Molecular Medicine Finland (FIMM), University of Helsinki, Helsinki, Finland; Johns Hopkins School of Medicine, United States of America

## Abstract

**Background:**

Current treatment options for castration- and treatment-resistant prostate cancer are limited and novel approaches are desperately needed. Our recent results from a systematic chemical biology sensitivity screen covering most known drugs and drug-like molecules indicated that aldehyde dehydrogenase inhibitor disulfiram is one of the most potent cancer-specific inhibitors of prostate cancer cell growth, including TMPRSS2-ERG fusion positive cancers. However, the results revealed that disulfiram alone does not block tumor growth *in vivo* nor induce apoptosis *in vitro*, indicating that combinatorial approaches may be required to enhance the anti-neoplastic effects.

**Methods and Findings:**

In this study, we utilized a chemical biology drug sensitivity screen to explore disulfiram mechanistic details and to identify compounds potentiating the effect of disulfiram in TMPRSS2-ERG fusion positive prostate cancer cells. In total, 3357 compounds including current chemotherapeutic agents as well as drug-like small molecular compounds were screened alone and in combination with disulfiram. Interestingly, the results indicated that androgenic and antioxidative compounds antagonized disulfiram effect whereas inhibitors of receptor tyrosine kinase, proteasome, topoisomerase II, glucosylceramide synthase or cell cycle were among compounds sensitizing prostate cancer cells to disulfiram. The combination of disulfiram and an antiangiogenic agent sunitinib was studied in more detail, since both are already in clinical use in humans. Disulfiram-sunitinib combination induced apoptosis and reduced androgen receptor protein expression more than either of the compounds alone. Moreover, combinatorial exposure reduced metastatic characteristics such as cell migration and 3D cell invasion as well as induced epithelial differentiation shown as elevated E-cadherin expression.

**Conclusions:**

Taken together, our results propose novel combinatorial approaches to inhibit prostate cancer cell growth. Disulfiram-sunitinib combination was identified as one of the potent synergistic approaches. Since sunitinib alone has been reported to lack efficacy in prostate cancer clinical trials, our results provide a rationale for novel combinatorial approach to target prostate cancer more efficiently.

## Introduction

Prostate cancer is the most frequent cancer and the second leading cause of cancer deaths in male population in the Western world [1]. Since most prostate cancer patients eventually become resistant to currently existing drugs such as anti-androgens and later also to cytotoxic agents, novel drugs and combinatorial approaches are needed. We have recently performed a chemical biology compound screen to systemically test the sensitivities of 4910 known drugs and drug-like small molecules in non-malignant and malignant prostate cancer cells [2]. Aldehyde dehydrogenase (ALDH) inhibitor disulfiram was among four cancer selective inhibitors identified blocking the growth of cultured TMPRSS2-ERG fusion positive VCaP cells at nanomolar concentration as well as reducing VCaP xenograft growth *in vivo* [2]. Recently, the growth inhibitory potential of disulfiram in prostate cancer has been confirmed in an independent high-throughput compound screen *in vitro* and xenograft studies *in vivo* [3], [4].

Disulfiram is an ALDH inhibitor that has been long-term used as an alcohol deterrent in the clinics. In addition to prostate cancer, disulfiram has also been shown to have anticancer effect in breast, myeloma, leukemia, lung cancer, cervical adenocarcinoma, melanoma, neuroblastoma and colorectal cancer [5]–[11]. Currently, disulfiram is in Phase I clinical trials in metastatic melanoma, in hormone refractory cancers with lung and liver metastases (www.clinicaltrials.gov, identifiers NCT00256230 and NCT00742911) as well as in prostate cancer (identifier: NCT01118741). In cultured prostate cancer cells, disulfiram induces oxidative stress, reduces ALDH and DNA methyltransferase (DNMT) activities as well as inhibits DNA replication [2], [4], [12]. In breast cancer, disulfiram and copper co-exposure inhibits NF-kB activity, increases reactive oxygen species and the number of cancer stem cells (CSC) [13]. Moreover, inhibition of ALDH activity has been suggested as a potential mean to reduce cancer stem cells and to overcome drug resistance [14]. Our previous results indicated that although disulfiram reduced VCaP cell xenograft growth approximately by 40%, it was not able to block it [2]. Similar results have been obtained in in human bone metastatic LNCaP C4-2B xenografts [4]. In addition, disulfiram exposure alone was not sufficient to induce apoptosis in prostate cancer cells [2]. Thus, in this study, we performed a combinatorial sensitivity screen in ERG positive prostate cancer cells to explore disulfiram mechanism of action in more detail. Moreover, the aim was to identify potential agents synergizing with disulfiram in prostate cancer cells. In total, 3357 compounds including current chemotherapeutic agents and drug-like small molecular compounds were studied alone and in combination with disulfiram. The molecular and phenotypic alterations were explored with one of the most potent disulfiram sensitizer, sunitinib.

## Materials and Methods

### Cells

The *TMPRSS2-ERG* gene fusion and AR positive prostate carcinoma cell line VCaP was received from Drs. Adrie van Bokhoven (University of Colorado Health Sciences Center, Denver, Colorado) and Kenneth Pienta (University of Michigan, Michigan) and were grown in Dulbecco’s Modified Eagle’s Medium [15]. Prostate carcinoma PC-3 cells were purchased from American Type Culture Collection (LGC Promochem AB, Borås, Sweden) and grown according to provider’s instructions.

**Figure 1:**
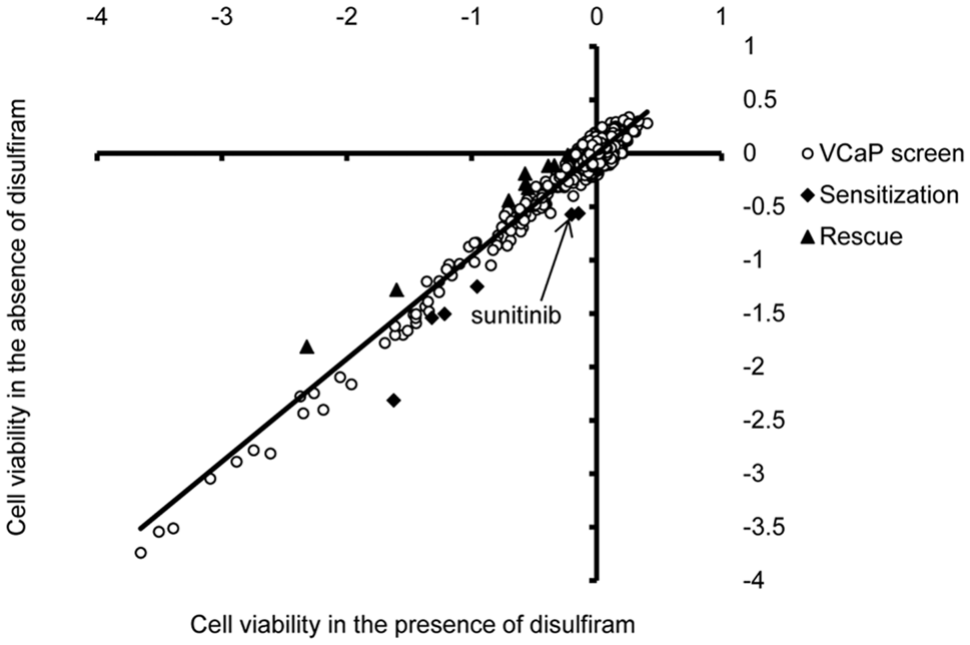
Combinatorial high-throughput cell viability screen to identify disulfiram modulating compounds. Loess-normalized CellTiter-Glo results with 3357 compounds screened in the absence (y-axis) and presence (x-axis) of disulfiram (EC_50_ 90 nM) in VCaP prostate cancer cells. Each dot represents result obtained with one compound. Data points qualifying as disulfiram sensitizing (squares below the trendline) and rescuing (triangles above the trendline) compounds are indicated. Result with sunitinib is indicated by an arrow.

### Compounds

Disulfiram was purchased from Fluka (Munich, Germany) and diluted in DMSO. Sunitinib was purchased from LC Laboratories (Woburn, USA) and diluted in DMSO.

**Table 1:** Compounds sensitizing the effect of disulfiram.

Compound	Concentration inthe screen	Description	Inhibition of cellviability alone (%)	Inhibition of cell viability in combination with DSF EC_50_ (%)
Bortezomib	2.6 mg/ml	Proteasome inhibitor	−68	−80
CGP-74514A hydrochloride	1 µM	Cyclin-dependent kinase 1(Cdk1) inhibitor	−10	−32
Epirubicin hydrochloride	10 µM	Topoisomerase II inhibitor	−57	−65
Phorbol 12-myristate 13-acetate	1 µM	Protein kinase C (PKC) activator	−60	−66
Sunitinib	10 µM	Receptor tyrosine kinase(RTK) inhibitor	−15	−35
Threo-1-Phenyl-2-decanoylamino-3-morpholino-1-propanol hydrochloride	0.1 µM	Glucosylceramide synthase(GCS) inhibitor	−49	−58

### High-throughput Compound Sensitivity Screen

A high-throughput compound sensitivity screen with the library of 3357 compounds alone and in combination with disulfiram was performed in VCaP cells. The library included current chemotherapeutics and small molecular compounds of commercial compound libraries LOPAC (1,280 existing Food and Drug Administration–approved drugs and other compounds with pharmacologically relevant structures; 1 and 0.1 µmol/L), Microsource Spectrum (2,000 compounds including most of the known drugs and other bioactive compounds and natural products; 1 and 0.1 µmol/L), and an inhouse library (77 experimental compounds; 10, 1, and 0.1 µmol/L). In the screen, EC_50_ value of disulfiram (90 nM) was used. The cell viability was determined after 3-day incubation using a CellTiter-Glo (CTG) fluorescent cell viability assay (Promega, Inc.). The cell viability results were normalized using a loess method as previously described [2]. The compounds that qualified as hits inhibited cell viability by at least three standard deviations from the median of the DMSO controls.

**Table 2:** Compounds rescuing the effect of disulfiram.

Compound	Concentrationin the screen	Description	Inhibition of cellviability alone (%)	Inhibition of cell viability in combination with DSF EC_50_ (%)
4-Androstene-3,17-dione	1 µM	Testosterone precursor andmetabolite with androgenicactivity	−24	−8
5-alpha-Androstane-3-alpha,17-beta-diol	1 µM	Testosterone metabolite	−33	−12
Androsterone	1 µM	Anabolic steroid	−21	−8
Astaxanthin	1 µM	Antioxidant	−15	−1
Cetuximab	0.2 mg/ml	Epidermal growth factorreceptor (EGFR) inhibitor	−39	−26
Dequalinium analog, C-14 linker	1 µM	Protein kinase C-alpha(PKC-alpha) inhibitor	−80	−71
Tyrphostin AG 528	1 µM	Epidermal growth factorreceptor (EGFR) inhibitor	−32	−20
Vinorelbine ditartrate	10 mg/ml	Microtubule assembly inhibitor	−67	−59

### Cell Viability and Apoptosis Assays

Cell viability and apoptosis assays were performed on 384-well plates (Falcon). 2,000 cells per well were plated in 35 µl of their respective growth media and left to attach overnight. Compound dilutions were added to the cells in 15 µl and incubated for 48 h. Cell viability was determined using the CTG cell viability assay (Promega, Inc.). Induction of caspase-3 and 7 activities was detected with homogenous Apo-ONE assay (Promega, Madison, WI). Cell viability and apoptosis assays were then performed according to the manufacturer’s instructions. Briefly, in the cell viability assay, 25 µl of activated CTG reagent was added to each well, the plate was incubated for 30 min at RT/150 rpm and the luminescence signal (700 nm) was quantified using Envision Multilabel Plate Reader (Perkin-Elmer, Massachusetts, MA). For the apoptosis assay, 25 µl of media was taken out from each well and 25 µl of ApoONE reagent was added into each well. The plate was incubated for 2 hours at RT and the fluorometric signal (excitation FITC 499 nm, emission FITC 521 nm) was quantified using Envision Multilabel Plate Reader. The average luminescence or fluorometric signal from the six replicate compound treated wells were divided by the average signal of six DMSO vehicle control treated wells to determine fold changes.

**Figure 2:**
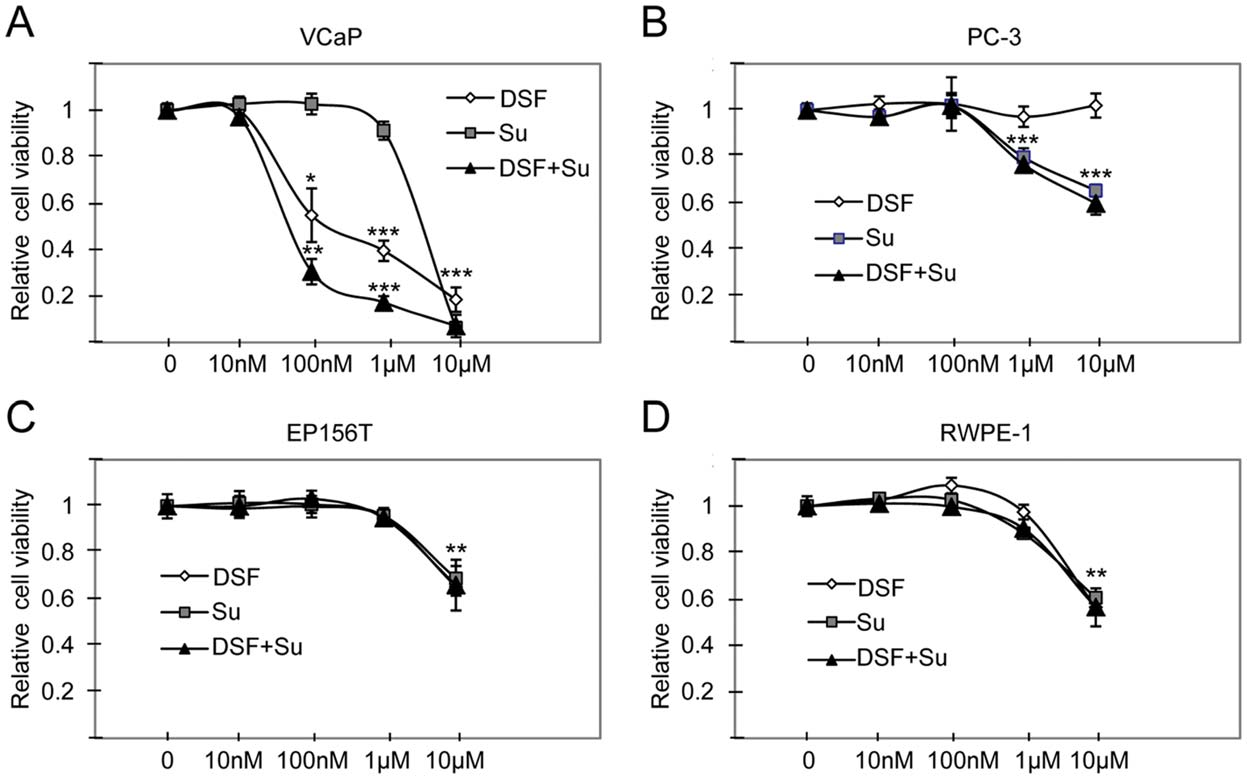
Illustration of disulfiram (DSF), sunitinib and disulfiram-sunitinib co-exposure induced effects on prostate cell viability. Relative cell viability results in A) VCaP and B) PC-3 prostate cancer cells as well as in non-malignant C) RWPE-1 and D) EP156T cells. Asterisks indicate the statistical significance: *, P<0.05; **, P<0.01; and ***, P<0.005.

### Statistical Analyses

The hit criteria in compound screen (score lower than −3 SD from the median) correspond to a P value of <0.01. Statistical analyses of all results were done by using the Student’s t-test. These results are presented as the mean ± SD. The following P values were used to show statistical significance: *, P<0.05; **, P<0.01; and ***, P<0.005.

### Determination of Combinatorial Drug Effects

The nature of interaction and the degree of synergy between disulfiram and sunitinib were analyzed using the combination index method [16]. The concentration dependence of antiproliferative effects was determined for both compounds, either alone or in combination. Fraction affected (Fa) was defined as the fraction of cells affected by the given concentration of compounds alone or in combination. Fa = 0 was determined based on DMSO control and Fa = 1 on staurosporine (1 µM) response (no viable cells left). The data was analyzed with Calcusyn software (Biosoft, Cambridge, UK), and the combination index (CI) was calculated from the median effect plots according to equation CI = (D)1/(DX)1+(D)2/(DX)2, where (DX)1 and (DX)2 are the concentrations of compounds D1 and D2 needed to produce a given level of antiproliferative effect when used alone, whereas (D)1 and (D)2 are their concentrations that produce the same effect when used in combination. A combination index of 0.9–1.1 indicates additive interaction, values below 0.9 indicate synergism, and values over 1.1 indicate antagonism.

**Figure 3:**
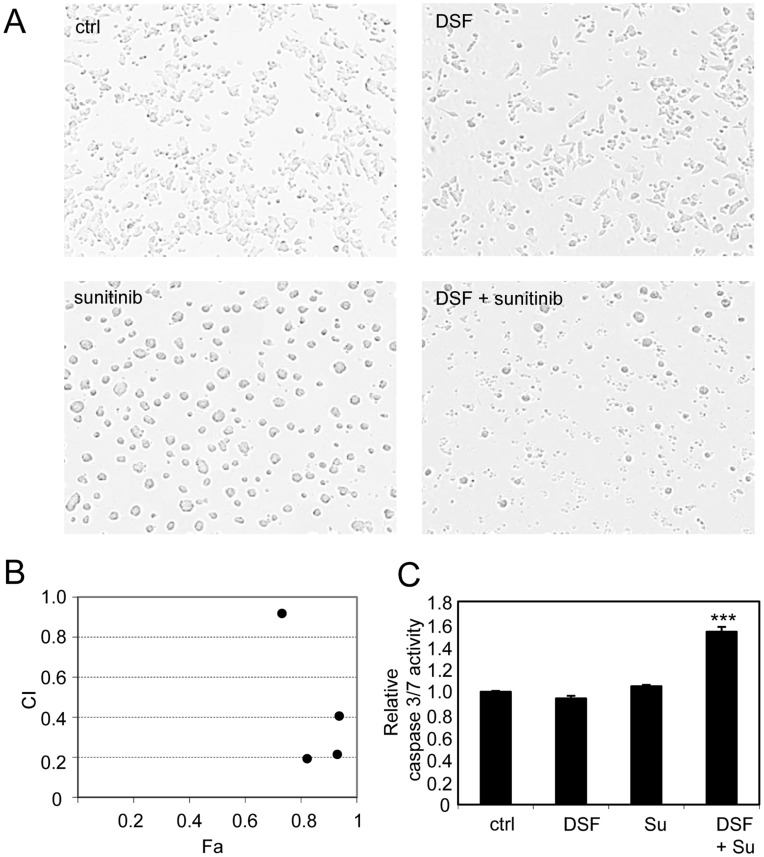
Sunitinib shows synergism with disulfiram in prostate cancer cells. A) Cell morphology in response to disulfiram (1 µM) and sunitinib (5 µM) exposures alone and in combination. B) Presentation of combination index (CI) and fraction of cells affected (Fa) by compound exposures in different concentrations (500 nM, 1 µM, 5 µM and 10 µM) in VCaP prostate cancer cells. CI values for each concentration: 500 µM: 0.92, 1 µM: 0.19, 5 µM: 0.21, 10 µM: 0.40. C) Caspase 3/7 activities in response to compound exposures. Asterisks indicate the statistical significance: ***, P<0.005.

### RNA Extraction and Quantitative Reverse Transcriptase PCR

Total RNA was extracted from cultured cells using RNeasy (Qiagen) according to the manufacturer’s protocol. Reverse transcription using 500 ng of total RNA was performed using Applied Biosystems cDNA synthesis kit. TaqMan gene expression probes and primers from the Universal Probe Library (Roche Diagnostics, Espoo, Finland) were used to study androgen receptor (AR), prostate specific antigen (PSA), ERG, MYC and β-actin mRNA expression. Primer sequences are listed in Table S1. Real-time quantitative PCR was performed using ABI Prism 7900 (Applied Biosystems, Foster City, CA). Quantitation was carried out using the _ΔΔ_CT method with RQ manager 1.2 software (Applied Biosystems). β-actin was used as an endogenous control. Average expression of the DMSO exposed control samples was considered for the calculation of the fold changes. Two to four replicate samples were studied for quantitation of mRNA expression.

### Western Blot Analysis and Subcellular Proteome Extraction

For protein extraction and Western blot analysis, VCaP cells were plated at 70% confluency and left to attach over night before treatments. Whole-cell lysates were prepared using lysis buffer (62.5 mM Tris, 1% SDS, 5%, β-mercaptoethanol, 10% glycerol and bromophenol blue). Three replicative samples were studied for quantitation of protein expression. Specific antibodies recognizing AR (1∶1000 dilution, mouse monoclonal, Labvision, Fremont, CA) or PSA (1∶1000, rabbit polyclonal, DakoCytomation, Denmark) were used. β-actin (1∶4000, mouse monoclonal, Sigma) was used as a loading control. Signals were detected with 1∶4000 dilutions of appropriate HRP-conjugated secondary antibodies (all from Invitrogen Molecular Probes, Carlsbad, CA) followed by visualization with the enhanced chemiluminescence reagent (Amersham Biosciences, Little Chalfont, UK). The obtained signals were densitometrically analyzed with GeneTools software (SynGene, Synoptics Ltd, Cambridge, UK).

**Figure 4:**
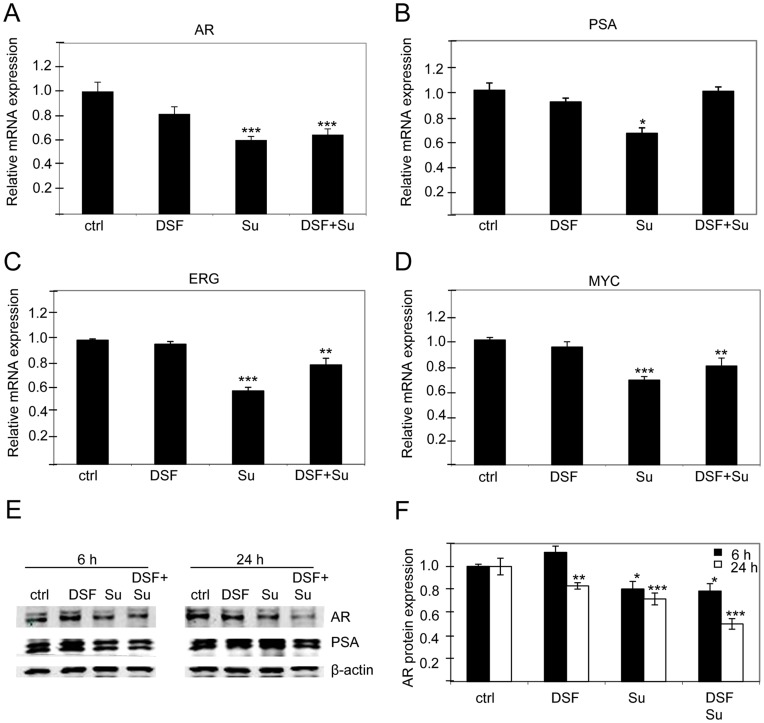
Disulfiram-sunitinib co-exposure reduces AR signalling in VCaP prostate cancer cells. The expression of A) AR, B) PSA, C) ERG, D) MYC mRNA in response to disulfiram (DSF) and sunitinib (Su) exposures alone and in combination. E) AR and PSA protein expression in response to compound exposures alone and in combination. F) Quantification of AR protein expression in response to 6- and 24 h compound exposures. Asterisks indicate the statistical significance: *, P<0.05; **, P<0.01; and ***, P<0.005.

**Figure 5:**
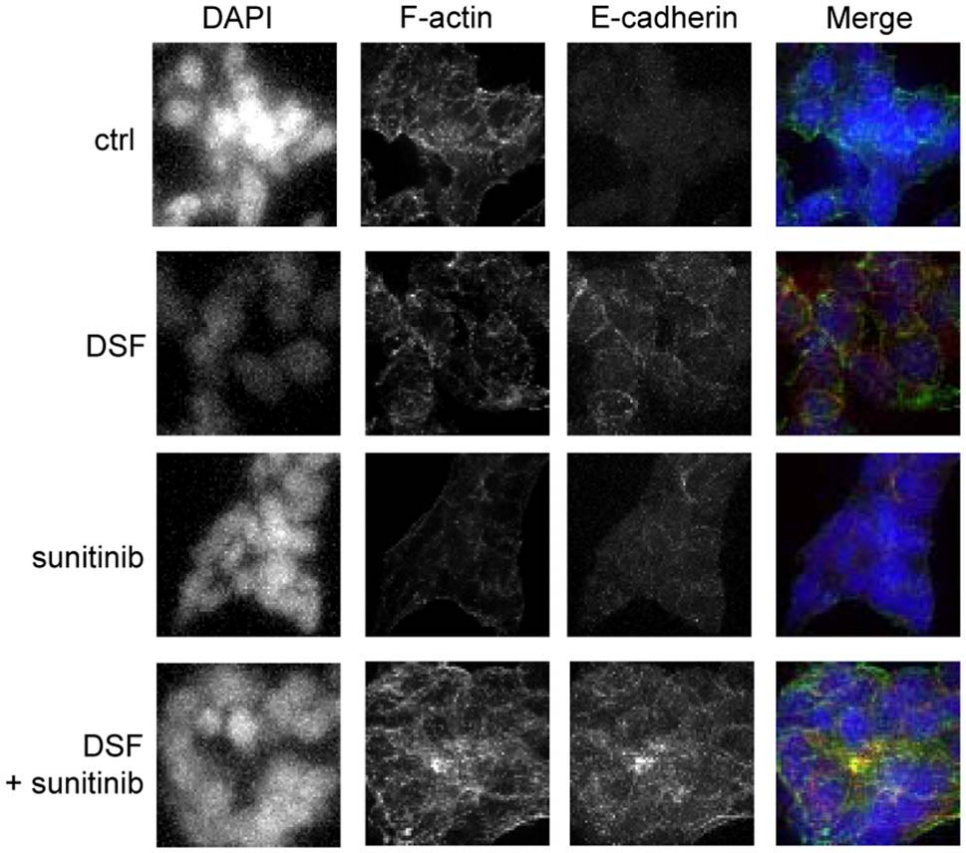
Disulfiram-sunitinib combination reduces E-cadherin expression. E-cadherin (red) and F-actin (yellow) expressions in response to compound treatments. DNA was stained with DAPI (blue).

### Immunofluorescence

Immunofluorescence staining of VCaP cells was carried out as previously described [12]. Images were taken with 63× magnification using a Zeiss Axiovert 200 M fluorescence microscope (Carl Zeiss AG, Oberkochen, Germany).

### Wound Healing Assay

The effect of disulfiram (1 µM) and sunitinib (5 µM) alone and in combination on prostate cancer cell migration was studied using a wound-healing assay. PC-3 cells were plated on 96-well plates (Essen ImageLock, Essen Instruments, Birmingham, UK) and a wound was scratched with wound scratcher (Essen Instruments). Compounds and appropriate controls were added immediately after wound scratching and wound confluence was monitored with Incucyte Live-Cell Imaging System and software (Essen Instruments). Wound closure was measured every hour for 24 h by comparing the mean relative wound density of three biological replicates in each experiment.

**Figure 6:**
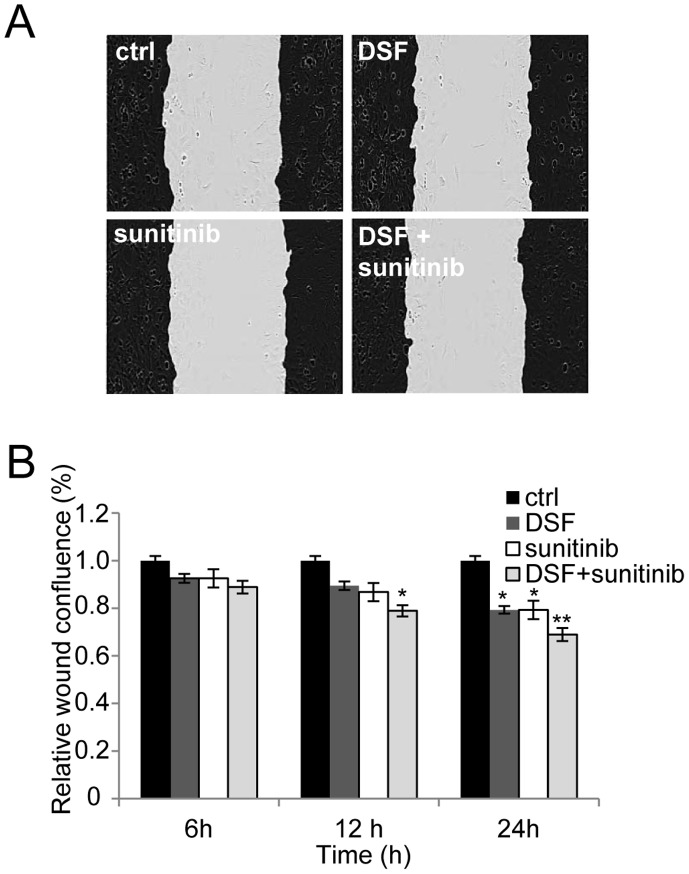
The effect of disulfiram and sunitinib cotreatment on PC-3 cell migration. Cells were automatically imaged once every hour after wound scratching. Wound closure effect was calculated as wound confluence in response 6-, 12- and 24 h exposures of the compounds A) Wound healing in response to compound exposures for 24 hours. Black area represents the wound edges in the beginning of the assay. B) Quantification of cells entering the wound area. Asterisks indicate the statistical significance: *, P<0.05; **, P<0.01; and ***, P<0.005.

**Figure 7:**
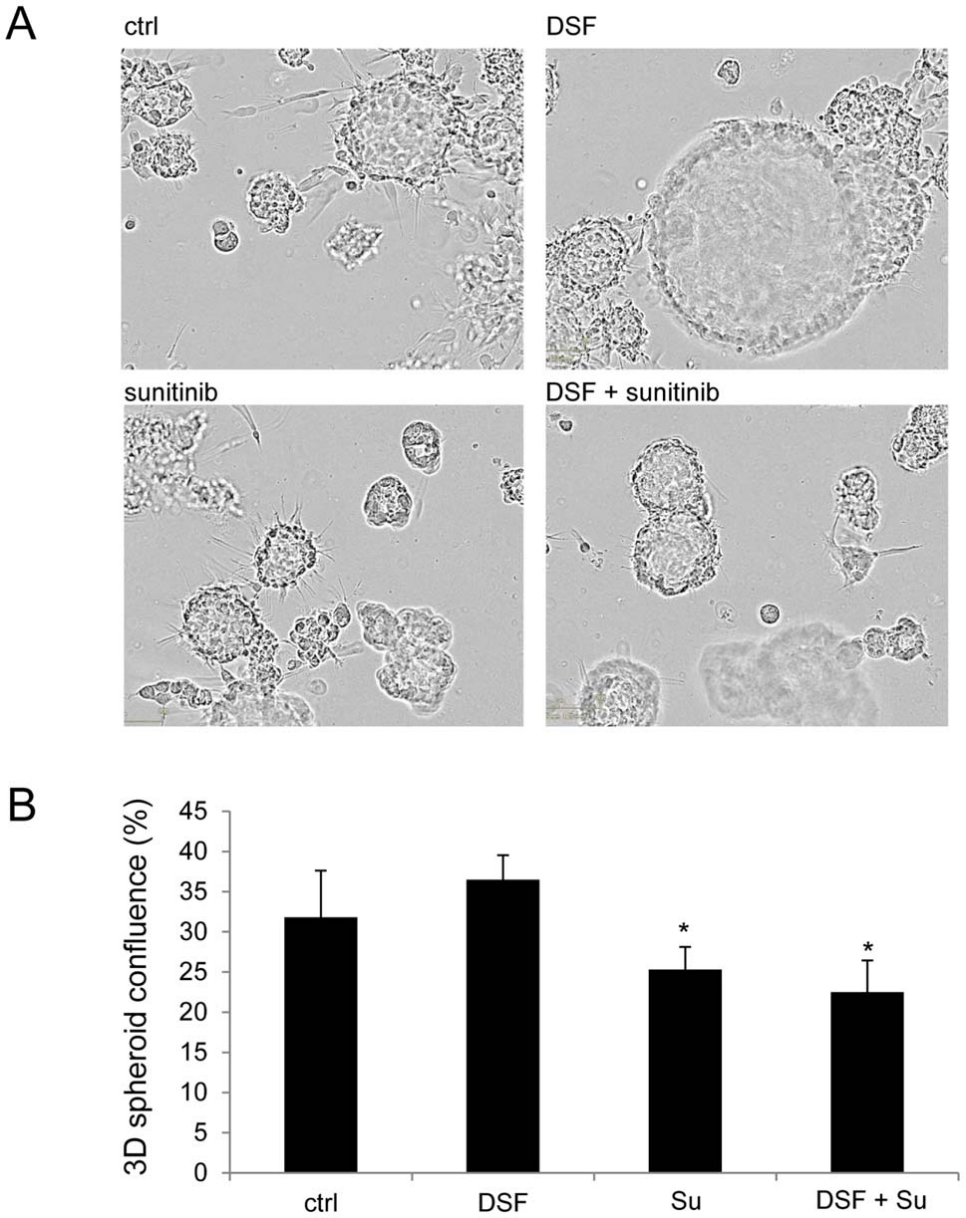
Disulfiram-sunitinib combination reduces PC-3 spheroid growth and invasion. A) Cell morphology in 3D spheroid assay in response to compound exposures. B) The area of cells in the Incucyte images (% of total area) in response to compound treatments. Asterisks indicate the statistical significance: *, P<0.05.

### 3D Assay

Cells were cultured in 3D on Matrigel on uncoated Angiogenesis μ-slides (Ibidi Gmbh, Germany). The bottom wells were filled with 10 µl of Matrigel (50%) in culture medium and incubated for 30 min in 37°C. The cells (1000 cells/well) were then plated and let to attach for 1–2 hours in 37°C. The second layer of Matrigel in culture medium (25%) was added and the plates were incubated in 37°C. Disulfiram (1 µM), sunitinib (5 µM) or disulfiram-sunitinib combination was added after 4 days of incubation and maintained for up to 7 days. Spheroids were monitored in real-time by live-cell imaging (Incucyte, Essen Instruments; 10× objective), acquiring 1 image/h. The area of 3D structures in the images was compared to the total image area (in percentages) to quantitate potential effects of the compounds on cell growth.

## Results

### Chemical Biology compound Sensitivity Screen Identifies Synergistic Agents with Disulfiram

Chemical biology compound screen approach was utilized to study the mechanism of disulfiram reduced cell viability in prostate cancer cells and to explore potential synergistic interactions of disulfiram and screened compounds. Library of 3357 compounds including most of the known drugs and drug-like small molecular compounds was screened alone and in combination with disulfiram in VCaP prostate cancer cells. The cell viability results in the absence (DMSO control) or presence of disulfiram (EC_50_, 90 nM) were compared. As expected, several compounds inhibited VCaP cell growth (Fig. 1). However, only 15 compounds showed a combination effect with disulfiram. In total, six compounds sensitised VCaP cells to disulfiram: threo-1-Phenyl-2-decanoylamino-3-morpholino-1-propanol hydrochloride, bortezomib, CGP-74514A hydrochloride, epirubicin hydrochloride, phorbol 12-myristate 13-acetate and sunitinib (Table 1). In contrast, 9 compounds rescued disulfiram induced antiproliferative effect (Table 2). Interestingly, these compounds included androgenic compounds 4-Androstene-3,17-dione, 5-alpha-Androstane-3-alpha,17-beta-diol and androsterone as well as antioxidant astaxanthin. Thus, the results indicate that disulfiram reduced cell proliferation may be antagonized with androgen activation or antioxidants. Moreover, PKC activator phorbol 12-myristate 13-acetate was among drugs sensitizing disulfiram effect and PKC inactivator dequalinium analog, C-14 linker, was among drugs rescuing disulfiram effect, indicating that PKC may play a role in disulfiram response. Furthermore, disulfiram effect may be potentiated via inhibition of receptor tyrosine kinases, proteasome, topoisomerase II, glucosylceramide synthase or cell cycle (Table 1) whereas inhibition of epidermal growth factor receptor seems not to be a potent strategy to enhance the effect of disulfiram (Table 2).

### Sunitinib and Disulfiram Cotreatment Show Synergism

Interestingly, sunitinib, an anti-angiogenic agent, potentiated the disulfiram induced growth-inhibitory effect. Sunitinib inhibits the activity of multiple tyrosine kinases such as VEGFR-1, -2 and -3, PDGFR-α and -β, c-Kit, Ret and Flt-3 [17]. It has been shown to have anti-neoplastic activities in a variety of malignancies such as hepatocellular cancer, pancreatic neuroendocrine tumors, and non-small cell lung cancer. Sunitinib is licenced to metastatic renal cell carcinoma and gastrointestinal tumors [18]–[20]. In prostate cancer, sunitinib reduces significantly the growth of castration-resistant prostate cancer, both in preclinical and clinical settings [21], [22]. However, a recent phase III study of sunitinib in prostate cancer failed due to lack of efficacy in castration-resistant prostate cancer (identifier: NCT00676650). Since sunitinib has already been studied in clinical trials in prostate cancer patients, sunitinib-disulfiram combination was selected for further *in vitro* studies.

To validate the combinatorial anti-proliferative effect of sunitinib in prostate cancer cells, the effect of disulfiram and sunitinib alone and in combination was studied in VCaP and PC-3 prostate cancer cells. The results indicated that disulfiram and sunitinib co-exposure reduced VCaP cell viability more than either of the compounds alone (Fig. 2A). In PC–3 cells, the anti-proliferative effect was seen only at higher concentrations in response to sunitinib or disulfiram-sunitinib co-exposure (Fig. 2B). To identify whether the synergism in VCaP cells is caused simply due to increased cytotoxicity, the effect of disulfiram, sunitinib or disulfiram-sunitinib co-exposure was studied in non-malignant RWPE-1 and EP156T prostate epithelial cells. The results showed that cell viability was decreased only at highest (10 µM) concentration in RWPE-1 and EP156T cells (Fig. 2C and D), indicating that the increase in overall cell toxicity does not explain the combinatorial response in VCaP cells.

To compare the effects of disulfiram (1 µM) and sunitinib (5 µM) alone and in combination on VCaP cell morphology, Incucyte live cell analysis was performed. Cells were exposed to compounds for 48 hours. Clear morphological changes were observed in response to all compound exposures (Fig. 3A). In particular, sunitinib caused cells to attach to each other since no individual cells were seen in sunitinib exposure cells. In response to disulfiram-sunitinib co-treatment, cells were also attached with each other, but there was clearly less viable cells left (Fig. 3A). Combination index (CI) was determined at various drug concentrations (500 nM, 1 µM, 5 µM and 10 µM) based on cell viability results in VCaP cells. The results indicated that disulfiram and sunitinib showed synergism at all concentrations tested (CI <1) (Fig. 3B). The lowest CI-values were seen in concentrations of 1 µM and 5 µM (CI 0.19 and 0.21). Sunitinib concentration of 5 µM was chosen for further combination experiments based on CI and cell viability results as well as previous sunitinib *in vitro* studies in prostate cancer cells [23]–[25].

### Sunitinib and Disulfiram Cotreatment Induces Apoptosis in Prostate Cancer Cells

To identify whether disulfiram and sunitinib exposure induces apoptosis, caspase 3 and 7 activities were determined by a quantitative fluorometric assay. Caspase activity was measured in response to disulfiram (1 µM) and sunitinib (5 µM) exposure for 48 hours alone and in combination in VCaP cells. Interestingly, neither disulfiram nor sunitinib alone was able to induce apoptosis. However, a significant induction of apoptosis was seen in response to disulfiram-sunitinib combination treatment (Fig. 3C). Taken together, sunitinib shows synergistic growth inhibitory effects with disulfiram and the combination of these two compounds induce apoptosis more than either of the compounds alone.

### Sunitinib Reduces the Expression of Androgen receptor (AR), Prostate Specific Antigen (PSA), ERG and MYC in ERG positive prostate cancer cells

To identify the first molecular changes in response to disulfiram and sunitinib, mRNA expression of prostate cancer oncogenes AR, PSA, ERG and MYC was studied in disulfiram (1 µM), sunitinib (5 µM) or disulfiram-sunitinib co-exposed VCaP cells at 3-hour time point. Interestingly, the results indicated that sunitinib significantly reduced AR, PSA, ERG and MYC levels (approximately by 40%) whereas disulfiram alone did not have major effect (Fig. 4A, B, C and D). The results with disulfiram are in accordance with our previous study [2]. However, there were no indications for a combinatorial effect of disulfiram and sunitinib on reducing the expression of these oncogenes at mRNA level.

To find out whether changes can be detected at protein level, AR was studied in response to longer exposures (6 and 24 hours) of disulfiram and sunitinib alone and in combination. Interestingly, only a slight reduction of AR was observed in response to sunitinib alone, and no decrease in AR protein expression was seen in response to disulfiram alone. However, a clear reduction of AR protein expression (20 and 50%) was observed in response to the combination exposure of disulfiram and sunitinib at 6- and 24-hour time points (Fig. 4E and F). Moreover, similar decrease in AR regulated PSA protein levels were observed (Fig. 4E). Taken together, these results indicate that sunitinib reduces androgen signalling in prostate cancer cells especially when combined with disulfiram. However, further analysis is needed to identify whether disulfiram and sunitinib act synergistically through androgen signalling.

### Disulfiram and Sunitinib Cotreatment Induces E-cadherin Expression

The results from microscopic cell morphology analysis suggested that VCaP cells were more attached to each other in response to either sunitinib or disulfiram and sunitinib co-exposure compared to disulfiram exposure alone (Fig. 3A). To identify whether these phenotypes were due to induction of cell adhesion molecule E-cadherin, immunochemical staining was performed. The results indicated that disulfiram and sunitinib cotreatment induced E-cadherin expression more than either of the compounds alone (Fig. 5). E-cadherin is commonly known marker for cancer cell differentiation and it is downregulated in invasive prostatic carcinoma [26]. We have previously shown that induction of E-cadherin expression is associated with reduced cell proliferation in ERG positive VCaP prostate cancer cells [27], [28]. These results indicate that morphological phenotype seen in response to sunitinib-disulfiram cotreatment correlates with elevated E-cadherin expression in VCaP prostate cancer cells.

### Disulfiram and Sunitinib Cotreatment Reduces Prostate Cancer Cell Migration

To study whether disulfiram and sunitinib cotreatment affects prostate cancer cell migration, live cell cell migration assay was done. In the assay, PC-3 cells were used as prostate cancer model, since VCaP cells do not migrate in this assay. The results indicated that disulfiram-sunitinib co-exposure reduced cell migration more than either one of the compounds alone (Fig. 6). The migration was reduced significantly in disulfiram and sunitinib co-exposed prostate cancer cells at 12- and 24-hour time points (by 20 and 30% compared to DMSO control). Significant decrease in cell migration was seen also in disulfiram and sunitinib exposed cells at 24-hour time point (Fig. 6B). PC-3 cell confluence was not significantly decreased at these time points, indicating that the reduction of cell migration does not result due to reduced cell proliferation (Figure S1). Thus, the results showed that disulfiram-sunitinib co-exposure reduces prostate cancer cell migration more than either of the compounds alone.

### Disulfiram and Sunitinib Combination Reduces Prostate Cancer Cell Invasion in 3D Culture

The effect of disulfiram and sunitinib cotreatment was studied in PC-3 3D spheroid model [29]. The spheroids were grown on Matrigel for 4 days and disulfiram (1 µM) and sunitinib (5 µM) alone and in combination were added to the cells and the cell morphology was monitored for 7 days using live-cell imaging. The results are shown in Figure 7. Disulfiram alone was able to reduce cells from invading 3D structure, but it was not able to reduce the growth of the cells inside the lumen (Fig. 7A). In contrast, sunitinib treated spheroids were smaller while cell invasion was not blocked. Interestingly, the combination treatment reduced the amount of invasive protrusions as well as the size of the spheroids. The area of cells in the images (% of total area) in response to compound treatments for 7 days in 3D is presented in Figure 7B. Taken together, disulfiram-sunitinib cotreatment reduced prostate cancer cell invasion and growth in 3D spheroid model.

## Discussion

In this study, we utilized a chemical biology compound sensitizing screen to study aldehyde dehydrogenase (ALDH) inhibitor disulfiram mechanism of action and to identify potential synergistic agents for disulfiram in TMPRSS2-ERG positive prostate cancer cells. Total of 3357 compounds including current chemotherapeutics and small molecular compounds were screened alone and in combination with disulfiram and the synergistic mechanism for disulfiram sensitizer sunitinib was studied in more detail.

The results from the high-throughput combinatorial screen indicated that several androgenic compounds as well as an antioxidant astaxanthin were among compounds rescuing disulfiram induced anti-proliferative effect in prostate cancer cells. Our previous results indicated that disulfiram induced oxidative stress in prostate cancer cells [2]. Disulfiram increases ROS levels also in breast cancer cells [13]. Thus, the rescue effect of antioxidant astaxanthin in disulfiram exposed cells supports the previous results indicating that disulfiram reduced cell proliferation is mediated via induction of oxidative stress. The screening results also suggested that PKC plays a role in disulfiram response since PKC activator phorbol 12-myristate 13-acetate was among drugs sensitizing to disulfiram effect whereas PKC inactivator dequalinium analog, C-14 linker rescued disulfiram effect in the screen. Moreover, inhibition of receptor tyrosine kinases, proteasome, topoisomerase II, glucosylceramide synthase may be alternative ways to enhance disulfiram effect whereas inhibition of epidermal growth factor receptor has an opposite effect. One of the six compounds sensitizing VCaP cells to disulfiram induced anti-proliferative effect was antiangiogenic agent, tyrosine-protein kinase receptor inhibitor sunitinib. Sunitinib is an anticancer drug that is clinically used to treat metastatic renal cell carcinoma and gastrointestinal cancer patients. It has also been shown to have anti-neoplastic activity in hepatocellular cancer, pancreatic neuroendocrine tumors, and non-small cell lung cancer [18]–[20]. However, despite the promising results derived from *in vitro* and *in vivo* studies as well as from phase I and II clinical trials, the phase III trial in advanced castration-resistant prostate cancer was recently halted due to the lack of efficacy [21], [22] (identifier: NCT00676650). Since sunitinib had already been studied in prostate cancer clinical trials, the mechanism of disulfiram-sunitinib combination was studied in more detail.

In this study, disulfiram-sunitinib co-exposure was shown to reduce prostate cancer cell growth more than either of the compounds alone. Moreover, disulfiram-sunitinib cotreatment induced apoptosis whereas neither of the compounds alone promoted programmed cell death in TMPRSS2-ERG fusion positive VCaP prostate cancer cells. Our results suggest that reduced AR signalling may play a role in disulfiram-sunitinib co-treatment induced anti-proliferative response in prostate cancer cells since disulfiram alone had no effect on AR nor PSA expression while reduced AR and PSA levels were seen in sunitinib and disulfiram-sunitinib exposures. Interestingly, sunitinib has been known to reduce PSA levels in castration-resistant prostate cancer patients [30].

The cell phenotypical analysis revealed that disulfiram-sunitinib co-treatment induced cell attachment and epithelial cell differentiation marker E-cadherin protein expression. Moreover, combination exposure decreased prostate cancer cell migration and invasion in 2D and 3D cultures. Interestingly, in 3D prostate cancer cell invasion assay, disulfiram exposed cells kept their spheroidal conformation while luminal cells in the spheroids were able to proliferate. In contrast, sunitinib exposed spheroids were smaller than control exposed spheroids, but the cells were able to invade from the spheroid structures. The disulfiram-sunitinib combination exposure reduced cell number as well as caused formation of smaller spheroids which were not as invasive as control spheroids. Thus, our results suggest that disulfiram-sunitinib combination induces prostate cancer cell attachment and differentiation as well as reduces metastatic properties. Interestingly, sunitinib, as well as other antiangiogenic agents, have recently been reported to induce breast cancer chemoresistance through induction of cancer stem cells [31]. The authors suggested that the effectiveness of antiangiogenic agents could be potentiated with drugs that target cancer stem cells. Our results support this hypothesis, since the antiangiogenic agent sunitinib was potentiated by ALDH and cancer stem cell inhibitor disulfiram in prostate cancer cells. ALDH is a known marker of cancer stem cells and its inhibition reduces chemotherapy and radiation resistance in breast cancer [32]. Moreover, in a recent report of a hepatocellular carcinoma patient, sunitinib was shown to induce epithelial-to-mesenchymal transition (EMT) and thus caused chemotherapeutic resistance [33].

Taken together, the results of this study propose novel combinatorial means to target prostate cancer cells. Based on the validation results, we reveal disulfiram-sunitinib combination as a potent way to target prostate cancer cells. In addition to the high-throughput screen in 2D cell culture, the validation studies were done in 3D prostate cancer spheroid model recapitulating more the *in vivo* tumor growth than 2D cell culture. However, we emphasize that further *in vivo* preclinical and clinical studies are needed to validate these cell-based results. The advantage in this disulfiram-sunitinib combination approach is, that since both agents are already in human use and considered as potential prostate cancer inhibitors, translation of these results towards clinical trials could be relatively fast. Furthermore, our results provide further support for the hypothesis that antiangiogenic agents used in combination with drugs targeting cancer stem cells is a potent approach to prevent tumor growth and expansion.

## Supporting Information

Figure S1 **Relative cell confluence in the wound scratch assay.**
(TIFF)

Table S1 **Primer sequences used in quantitative reverse transcriptase PCRs.**
(XLS)
