# Supplementary figures and images for: Chemical Biology Drug Sensitivity Screen Identifies Sunitinib as Synergistic Agent with Disulfiram in Prostate Cancer Cells
Source: PLoS One. 2012 Dec 12;7(12):e51470. doi: 10.1371/journal.pone.0051470 (PMC3520796; doi:10.1371/journal.pone.0051470)

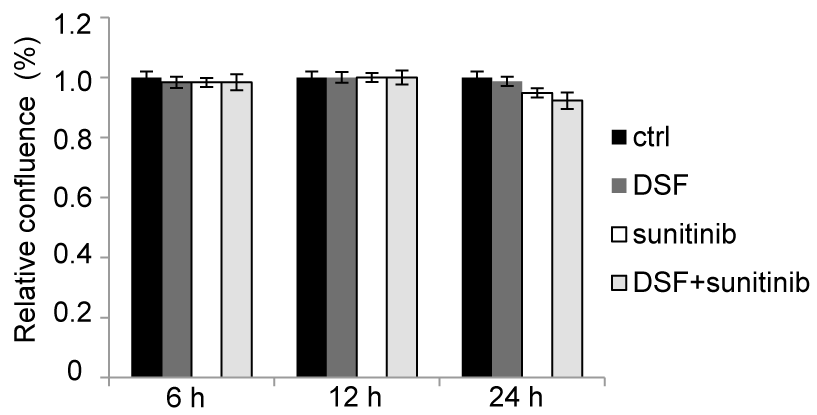

Supplement: Figure S1 — Relative cell confluence in the wound scratch assay. (TIFF) [file pone.0051470.s001.tiff]
